# Metacognitive asymmetries in visual perception

**DOI:** 10.1093/nc/niab005

**Published:** 2021-12-13

**Authors:** Matan Mazor, Rani Moran, Stephen M Fleming

**Affiliations:** Institute of Neurology, Wellcome Centre for Human Neuroimaging, University College London, London, UK; Institute of Neurology, Max Planck UCL Centre for Computational Psychiatry and Ageing Research, London, UK; Institute of Neurology, Wellcome Centre for Human Neuroimaging, University College London, London, UK; Institute of Neurology, Max Planck UCL Centre for Computational Psychiatry and Ageing Research, London, UK; Department of Experimental Psychology, University College London, London, UK

**Keywords:** absence, presence, metacognition

## Abstract

People have better metacognitive sensitivity for decisions about the presence compared to the absence of objects. However, it is not only objects themselves that can be present or absent, but also parts of objects and other visual features. Asymmetries in visual search indicate that a disadvantage for representing absence may operate at these levels as well. Furthermore, a processing advantage for surprising signals suggests that a presence/absence asymmetry may be explained by absence being passively represented as a default state, and presence as a default-violating surprise. It is unknown whether the metacognitive asymmetry for judgments about presence and absence extends to these different levels of representation (object, feature, and default violation). To address this question and test for a link between the representation of absence and default reasoning more generally, here we measure metacognitive sensitivity for discrimination judgments between stimuli that are identical except for the presence or absence of a distinguishing feature, and for stimuli that differ in their compliance with an expected default state.

## Introduction

At any given moment, there are many more things that are not there than things that are there. As a result, and in order to efficiently represent the environment, perceptual and cognitive systems have evolved to represent presences, and absence is implicitly represented as a default state (Oaksford and Chater 2001; Oaksford 2002). One corollary of this is that presence can be inferred from bottom-up sensory signals, but absence is never explicitly represented in sensory channels and must instead be inferred based on top-down expectations about the likelihood of detecting a hypothetical signal, had it been present. Experiments on human subjects accordingly suggest that representing absence is more cognitively demanding than representing presence, even in simple perceptual tasks, as is evident in slower reactions to stimulus absence than stimulus presence in near-threshold visual detection (Mazor *et al.* 2020), in a general difficulty to form associations with absence (Newman *et al.* 1980), and in the late acquisition of explicit representations of absence in development (e.g., Sainsbury 1971; Coldren and Haaf 2000; for a review on the representation of nothing see Hearst 1991).

**Figure 1.**
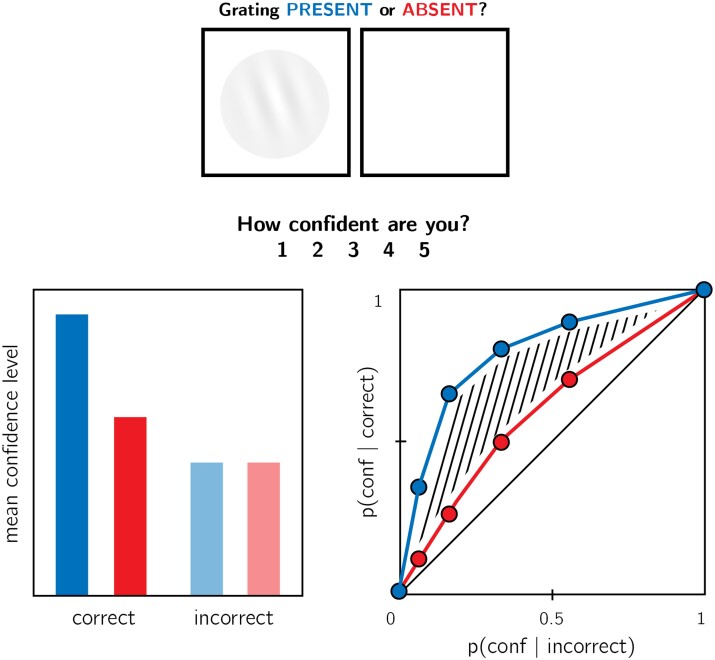
In visual detection, subjective confidence ratings following judgments about target absence are typically lower, and less correlated with objective accuracy than following judgments about target presence. Top panel: a typical detection experiment. The participant reports whether a visual grating was present or absent, and then rates their subjective decision confidence. Bottom left: typically, mean confidence in “yes” responses (blue) is higher than in “no” responses (red). This effect is much more pronounced in correct trials. Bottom right: the interaction between accuracy and response type on confidence (metacognitive asymmetry) manifests as a lower area under the response-conditional ROC curve for “no” responses compared with “yes” responses. Plots do not directly correspond to a specific dataset, but portray typical results in visual detection.

An overarching difficulty in representing absence may reflect the metacognitive nature of absence representations; to represent something as absent, one must assume that they would have detected it had it been present. In philosophical writings, this form of higher order, metacognitive inference-about-absence is known as *argument from epistemic closure*, or *argument from self-knowledge* (*If it was true, I would have known it*; De Cornulier 1988; Walton 1992). Strikingly, quantitative measures of metacognitive insight are consistently found to be lower for decisions about absence than for decisions about presence. When asked to rate their subjective confidence following near-threshold detection decisions, subjective confidence ratings following “target absent” judgments are commonly lower, and less aligned with objective accuracy, than following “target present” judgments ( Fig. 1; Kanai *et al.* 2010; Meuwese *et al.* 2014; Kellij *et al.* 2018; Mazor *et al.* 2020).

Metacognitive asymmetries have not only been observed for judgments about the presence or absence of whole physical objects and stimuli, but also for the presence or absence of cognitive variables such as memory traces. For instance, in recognition memory, subjects typically show poor metacognitive sensitivity for judgments about the absence of memories (such as when judging that they have not seen a study item before; Higham *et al.* 2009). Unlike the absence of a visual stimulus, the absence of a memory is not localized in space and does not correspond with a specific representation of “nothing”.

One way of conceptualizing these findings is that absence asymmetries emerge as a function of default reasoning—absences are considered the “default”, and information about perceptual or mnemonic presence is accumulated and tested against this default. For instance, an asymmetry may emerge in recognition memory because the presence of memories is actively represented, and the absence of memories is assumed as the default unless evidence is available for the contrary. In the same way, other visual features that are not typically treated as presences or absences may still be coded relative to a default—assuming one state unless evidence is available for the contrary (e.g., assuming that a cookie is sweet rather than salty). However, whether a metacognitive asymmetry in processing presence and absence generalizes to these more abstract violations of default expectations remains unknown. Here we set out to map out the structure of absence representations by testing for metacognitive asymmetries in the presence and absence of attributes at different levels of representation—from concrete objects, to visual features, to violations of default expectations.

Our choice of stimuli draws inspiration from visual search—a field where asymmetries are observed for a variety of stimulus types and features. In visual search, participants typically take longer to search for a target that is marked by the presence of a distinguishing feature, as compared to searching for a target that is marked by the absence of a feature relative to distractors (Treisman and Souther 1985; Treisman and Gormican 1988). Interestingly, *search asymmetries* have been demonstrated not only for the absence or presence of concrete physical features, but also for the presence or absence of deviations from a more abstract default state, which can be based on experience, culture, and contextual expectations (see Methods; Frith 1974; Von Grünau and Dubé 1994; Wang *et al.* 1994; Gandolfo and Downing 2020). Of special interest for our study are these latter asymmetries due to expectation violations, and their relation with asymmetries induced by the presence or absence of local and global features. Observing a metacognitive asymmetry for expectation violations as well as for the presence and absence of objects features would support a strong link between the representation of absence and default reasoning, where differences in metacognitive sensitivity reflect differences in the processing of information that agrees or contrasts with the expected default state.

While traditional accounts interpreted visual search asymmetries as reflecting a qualitative advantage for the cognitive representation of presence (affording a parallel search in the case of feature-present search only; Treisman and Gormican 1988), other models attribute the asymmetry to differences in the distributions of perceptual signals already at the sensory level (Dosher *et al.* 2004; Vincent 2011). Similarly, in the case of metacognitive asymmetries, the idea that decisions about absence are qualitatively different from decisions about presence has been challenged by an excellent fit of simple models that assume unequal variance for the signal-present and signal-absent sensory distributions, a model that does not assume any qualitative difference between the two decisions (Kellij *et al.* 2018). Deciding between these model families is beyond the scope of this project. However, identifying metacognitive asymmetries for abstract cognitive variables such as familiarity could help refine these models, for instance by revealing that representing deviations from a default state is an overarching principle of cognitive organization, one that goes beyond specific features of visual perception.

## Materials and Methods

We report how we determined our sample size, all data exclusions (if any), all manipulations, and all measures in the study.

**Figure 2.**
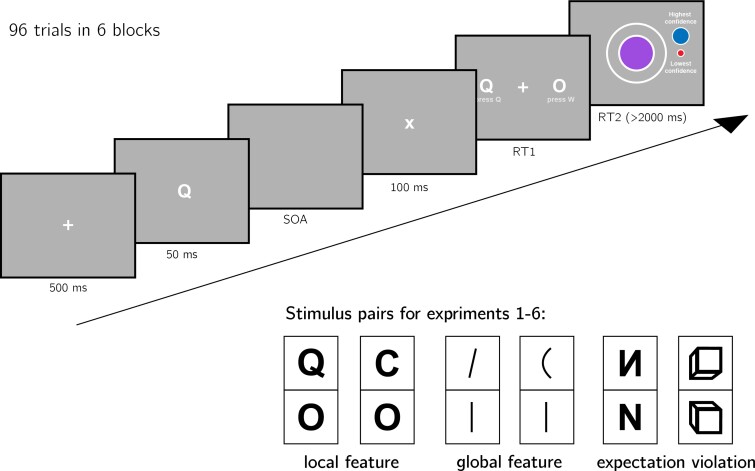
Response conditional ROC curves for the two discrimination responses. The area under the curve is a measure of metacognitive sensitivity. Bottom right inset: distributions of the area under the curve for the two responses, across participants. Overall, participants had lower metacognitive insight into the accuracy of their ‘O’ responses.

We will run six experiments, that will be identical except for the identity of the two stimuli $S_{1}$ and $S_{2}$. Our choice of stimuli for this study is based on the visual search literature. For some stimulus pairs $S_{1}$ and $S_{2}$, searching for one $S_{1}$ among multiple $S_{2}$s is more efficient than searching for one $S_{2}$ among multiple $S_{1}$s. Such *search asymmetries* have been reported for stimulus pairs that are identical except for the presence and absence of a distinguishing feature. Importantly, distinguishing features vary in their level of abstraction, from concrete *local features* (finding a Q among Os is easier than the inverse search; Treisman and Souther 1985), through *global features* (finding a curved line among straight lines is easier than the inverse search; Treisman and Gormican 1988), and up to the presence or absence of abstract *expectation violations* (searching for an upward-tilted cube among downward-tilted cubes is easier than the inverse search, in line with a general expectation to see objects on the ground rather than floating in space; Von Grünau and Dubé 1994). We treat these three types of asymmetries as reflecting a default-reasoning mode of representation, where the absence of features and/or the adherence of objects to prior expectations is tentatively accepted as a default by the visual system, unless evidence is available for the contrary (Treisman and Souther 1985; Treisman and Gormican 1988). In this study, we will test for metacognitive asymmetries for two stimulus features in each category, in six separate experiments with different participants (Fig. 2). For each of the following stimulus pairs, searching for $S_{1}$ among multiple instances of $S_{2}$ has been found to be more efficient than the inverse search:

1. **Local feature: Addition of a stimulus part**. *Q* and *O* will be used as $S_{1}$ and $S_{2}$, respectively (Treisman and Souther 1985).
2. **Local feature: Open ends**. *C* and *O* will be used as $S_{1}$ and $S_{2}$, respectively (Treisman and Souther 1985; Treisman and Gormican 1988; Takeda and Yagi 2000).
3. **Global feature: Curvature**. Curved and straight lines will be used as $S_{1}$ and $S_{2}$, respectively (Treisman and Gormican 1988).
4. **Global feature: Orientation**. Tilted and vertical lines will be used $S_{1}$ and $S_{2}$, respectively (Treisman and Gormican 1988).
5. **Expectation violation: Letter inversion**. Reversed and normal *N*s will be used as $S_{1}$ and $S_{2}$, respectively (Frith 1974; Wang *et al.* 1994).
6. **Expectation violation: Viewing angle**. Upward and Downward tilted cubes will be used as $S_{1}$ and $S_{2}$, respectively (Von Grünau and Dubé 1994).

The experiments will quantify participants’ metacognitive sensitivity for discrimination judgments between $S_{1}$ and $S_{2}$.

### Participants

The research complies with all relevant ethical regulations, and was approved by the Research Ethics Committee of University College London (study ID number 1260/003). Participants will be recruited via prolific, and will give informed consent prior to their participation. They will be selected based on their acceptance rate (>95%) and for being native English speakers. For each of the six experiments, we will collect data until we reach 106 included participants (after applying our pre-registered exclusion criteria). The entire experiment will take 15 min to complete. Participants will be paid £2 for their participation, equivalent to an hourly wage of £8.

### Procedure

Experiments were programmed using the jsPsych and P5 JavaScript packages (De Leeuw 2015; McCarthy 2015), and will be hosted on a JATOS server (Lange *et al.* 2015).

After instructions, a practice phase and a multiple-choice comprehension check, the main part of the experiment will start. It will comprise 96 trials separated into 6 blocks. Only the last five blocks will be analyzed.

On each trial, participants will make discrimination judgments on masked stimuli, and rate their subjective decision confidence on a continuous scale. After a fixation cross (500 ms), the target stimulus ($S_{1}$ or $S_{2}$) will be presented in the center of the screen for 50 ms, followed by a mask (100 ms). Stimulus onset asynchrony will be calibrated online in a 1-up-2-down procedure (Levitt 1971), with a multiplicative step factor of 0.9, and starting at 30 ms. Participants will then use their keyboard to make a discrimination judgment. Stimulus-key mapping will be counterbalanced between participants. Following response, subjective confidence ratings will be given on an analog scale by controlling the size of a colored circle with the computer mouse. High confidence will be mapped to a big, blue circle, and low confidence to a small, red circle. We chose a continuous (rather than a more typical discrete) confidence scale in order to ensure sufficient variation in confidence ratings within the dynamic range of individual participants. This variation is useful for the extraction of response conditional ROC curves. The confidence rating phase will terminate once participants click their mouse, but not before 2000 ms. No trial-specific feedback will be delivered about accuracy. In order to keep participants motivated and engaged, block-wise feedback will be delivered between experimental blocks about overall accuracy, mean confidence in correct responses, and mean confidence in incorrect responses.

### Randomization

The order and timing of experimental events will be determined pseudo-randomly by the Mersenne Twister pseudorandom number generator, initialized in a way that ensures registration time-locking (Mazor *et al.* 2019).

### Data Analysis

We will use R (Version 3.6.0; R Core Team 2019) and the R-packages *BayesFactor* (Version 0.9.12.4.2; Morey and Rouder 2018), *broom* (Version 0.5.6; Robinson and Hayes 2020), *cowplot* (Version 1.0.0; Wilke 2019), *dplyr* (Version 1.0.0; Wickham *et al.* 2020), *ggplot2* (Version 3.3.1; Wickham 2016), *lsr* (Version 0.5; Navarro 2015), *MESS* (Version 0.5.6; Ekstrøm 2019), *papaja* (Version 0.1.0.9942; Aust and Barth 2020), *pracma* (Version 2.2.9; Borchers 2019), *pwr* (Version 1.3.0; Champely 2020), and *tidyr* (Version 1.1.0; Wickham and Henry 2020) for all our analyses.

For each of the six stimulus pairs [$S_{1}$, $S_{2}$], we will test the following hypotheses:

1. **Hypothesis 1**: Subjective confidence is higher for $S_{1}$ responses than for $S_{2}$ responses. For each of the six stimulus pairs, we will test the null hypothesis that subjective confidence for $S_{1}$ responses is equal to or lower than subjective confidence for the feature-absent stimulus ($H_{o}:\mathrm{con}f_{S_{1}}\leq \mathrm{Con}f_{S_{2}}$).
2. **Hypothesis 2**: Metacognitive sensitivity, measured as the area under the response conditional ROC curves, is higher for $S_{1}$ responses than for $S_{2}$ responses. For each of the six stimulus pairs, we will test the null hypothesis that metacognitive sensitivity for $S_{1}$ responses is equal to or lower than metacognitive sensitivity for $S_{2}$ responses ($H_{o}:\mathrm{auRO}C_{S_{1}}\leq \mathrm{auRO}C_{S_{2}}$).
3. **Hypothesis 3**: Metacognitive sensitivity, measured as the area under the response conditional ROC curves, is higher for $S_{1}$ responses than for $S_{2}$ responses, to a greater extent than expected from an equivalent equal-variance SDT model. For each of the six stimulus pairs, we will test the null hypothesis that difference between metacognitive sensitivities for $S_{1}$ and $S_{2}$ responses is lower than the difference expected from an equal-variance SDT model with matched confidenec distributions, response bias, and sensitivity ($H_{o}:(\mathrm{auRO}C_{S_{1}}-\mathrm{auRO}C_{S_{2}})\leq ({\hat{\mathrm{auROC}}}_{S_{1}}-{\hat{\mathrm{auROC}}}_{S_{2}})$).
4. **Hypothesis 4**: $S_{1}$ responses are faster on average than $S_{2}$ responses. For each of the six stimulus pairs, we will test the null hypothesis that log-transformed response times for $S_{1}$ responses are equal to or higher than log-transformed response times for $S_{2}$ responses ($H_{o}:\log(RT_{S_{1}})\geq \log(RT_{S_{2}})$).

Hypotheses 1 and 2 correspond to the effects of stimulus type on metacognitive bias and metacognitive sensitivity, respectively. Although these two measures are theoretically independent, both bias and sensitivity are found to vary between detection “yes” and “no” responses.

Based on pilot data and previous experiments examining near-threshold perceptual detection and discrimination, we do not expect a response bias (such that the probability of responding $S_{1}$ is significantly different from 0.5 across participants). However, such a response bias, if found, may bias our metacognitive asymmetry estimates as measured with response-conditional ROC curves. Hypothesis 3 is designed to confirm that metacognitive asymmetry is higher than that expected from an equivalent equal-variance SDT model with the same response bias, sensitivity, and distribution of confidence ratings in incorrect responses as in the actual data. We will interpret conflicting results for Hypotheses 2 and 3 as evidence for a metacognitive asymmetry that is driven or masked by a response bias.

Hypothesis 4 is motivated by two observations from previous studies. First, detection “yes” responses are faster than detection “no” responses (Mazor *et al.* 2020). And second, when participants are not under strict time pressure, reaction time inversely scales with confidence (Henmon 1911; Pleskac and Busemeyer 2010; Calder-Travis *et al.* 2020). Based on these findings, if $S_{1}$ and $S_{2}$ responses are similar to detection “yes” and “no” responses not only in explicit confidence judgments, but also in response times, we should also expect a response time difference for these stimulus pairs.

### Dependent variables and analysis plan

Response conditional ROC curves will be extracted by plotting the empirical cumulative distribution of confidence ratings for correct responses against the same cumulative distribution for incorrect responses. This will be done separately for the two responses $S_{1}$ and $S_{2}$, resulting in two curves. The area under the response-conditional ROC curve is a measure of metacognitive sensitivity (Fleming and Lau 2014). The difference between the areas for the two responses is a measure of metacognitive asymmetry (Meuwese *et al.* 2014). This difference will be used to test Hypothesis 2.

In order to test hypothesis 3, SDT-derived response-conditional ROC curves will be plotted in the following way. For each response, we will plot the empirical cumulative distribution for incorrect responses on the x axis against the cumulative distribution for correct responses that would be expected in an equal-variance SDT model with matching sensitivity and response bias on the y axis. The difference between the areas of these theoretically derived response-conditional ROC curves will be compared against the difference between the true response-conditional ROC curves.

For visualization purposes only, confidence ratings will be divided into 20 bins, tailored for each participant to cover their dynamic range of confidence ratings.

For each of the six experiments, Hypotheses 1–4 will be tested using a one tailed *t*-test at the group level with α=0.05. The summary statistic at the single subject level will be difference in mean confidence between $S_{1}$ and $S_{2}$ responses for Hypothesis 1, difference in area under the response-conditional ROC curve between $S_{1}$ and $S_{2}$ responses (ΔAUC) for Hypothesis 2, difference in ΔAUC between true confidence distributions and SDT-derived confidence distributions for hypothesis 3, and difference in mean log response time between $S_{1}$ and $S_{2}$ responses for Hypothesis 4.

In addition, a Bayes factor will be computed using the BayesFactor R package (Morey *et al.* 2015) and using a Jeffrey-Zellner-Siow (Cauchy) Prior with an rscale parameter of 0.65, representative of the similar standardized effect sizes we observe for Hypotheses 1–4 in our pilot data.

We will base our inference on the resulting Bayes Factors.

### Statistical power

Statistical power calculations were performed using the R-pwr packages pwr (Champely 2020).

1. Hypothesis 1 (MEAN CONFIDENCE): With 106 participants, we will have a statistical power of 95% to detect effects of size 0.32, which is less than the standardized effect size we observed for confidence in our pilot sample (d=0.66).
2. Hypothesis 2 (METACOGNITIVE ASYMMETRY): With 106 participants, we will have a statistical power of 95% to detect effects of size 0.32, which is less than the standardized effect size we observed for metacognitive sensitivity in our pilot sample (d=0.73).
3. Hypothesis 3 (METACOGNITIVE ASYMMETRY: CONTROL): With 106 participants, we will have a statistical power of 95% to detect effects of size 0.32, which is less than the standardized effect size we observed for metacognitive sensitivity, controlling for response bias, in our pilot sample (d=0.81).
4. Hypothesis 4 (RESPONSE TIME): With 106 participants, we will have a statistical power of 95% to detect effects of size 0.32, which is less than the standardized effect size we observed for response time in our pilot sample (d=0.61).

Finally, in case that the true effect size equals 0, a Bayes Factor with our chosen prior for the alternative hypothesis will support the null in 95 out of 100 repetitions, and will support the null with a $BF_{01}$ higher than 3 in 79 out of 100 repetitions. In a case where the true effect size is sampled from a Cauchy distribution with a scale factor of 0.65, a Bayes Factor with our chosen prior for the alternative hypothesis will support the alternative hypothesis in 76 out of 100 repetitions, support the alternative hypothesis with a $BF_{10}$ higher than 3 in 70 out of 100 repetitions, and support the null hypothesis with a $BF_{01}$ higher than 3 in 15 out of 100 hypotheses (based on an adaptation of simulation code from Lakens 2016).

### Rejection criteria

Participants will be excluded for performing below 60% accuracy, for having extremely fast or slow reaction times (below 250 ms or above 5 s in more than 25% of the trials), and for failing the comprehension check. Finally, for type-2 ROC curves to be generated, some responses must be incorrect. Thus, only participants who committed at least two errors of each error type (e.g., mistaking a *Q* of *O* and mistaking an *O* for *Q*), will be included.

Trials with response time below 250 ms or above 5 s will be excluded.

## Supplementary data

Supplementary data is available at *NCONSC Journal* online.

## Data Availability

All raw data will be made fully available on OSF and on the study’s GitHub respository: https://github.com/matanmazor/asymmetry. Pilot data is available at: https://github.com/matanmazor/asymmetry/blob/master/Experiments/Q_in_O/results/pilot/jatos_results_batch3.csv

## Code Availability

All analysis code will be openly shared on the study’s GitHub repository: https://github.com/matanmazor/asymmetry. For complete reproducibility, the RMarkdown file used to generate the final version of the manuscript, including the generation of all figures and extraction of all test statistics, will be available on our GitHub repository.

*Conflict of interest statement*. None declared.

## Funding

The Wellcome Centre for Human Neuroimaging is supported by core funding from the Wellcome Trust (203147/Z/16/Z). S.M.F. is supported by a Sir Henry Dale Fellowship jointly funded by the Wellcome Trust and the Royal Society (206648/Z/17/Z).

## Supplementary Material

niab005_Supp
