# Supplementary material for: Metacognitive asymmetries in visual perception
Source: Neurosci Conscious. 2021 Dec 13;2021(1):niab005. doi: 10.1093/nc/niab005 (PMC8216202; doi:10.1093/nc/niab005)
Supplement: niab005_Supp [file niab005_supp.zip › niab005_Supplementary_Data.docx]

# Supplementary information

# Pilot data and analysis

## Pilot Experiment

30 participants were recruited from Prolific for our pilot experiment. We followed a similar procedure to the one described in the Methods section, using *Q* and *O* as our stimuli. In this pilot study we also replicated the visual-search asymmetry for these stimuli. To keep the experiment short and participants engaged, participants only completed 32 discrimination trials. The experiment took about 13.5 minutes to complete. Subjects were paid £1.25 for their participation.

Mean accuracy was 0.80 in the discrimination task, 0.96 in the Q-in-O search task, and 0.95 in the O-in-Q search task. We excluded participants for performing below 70% accuracy in one or two of the search tasks, for performing below 60% accuracy in the discrimination task, for having extremely fast or slow reaction times in one or more of the tasks (below 250 milliseconds or above 5 seconds in more than 25% of the trials), and for failing the comprehension check. Overall we excluded 0 participants, leaving 30 participants for the main analysis.

## Visual search task: search asymmetry replication

Search time analysis was performed on correct trials with reaction time between 250 and 5000 milliseconds. Search slopes for the response time/set size function were extracted for each participant, task and response, and then subjected to a two-way analysis of variance. As expected, we observed significant effects for target identity (mean search slope for *Q-in-O* search: 18.93 ms/item; mean search slope for *O-in-Q* search: 54.89 ms/item; $F(1,116)=44.39$, $MSE=873.80$, $p<.001$) and response (mean search slope for target absent responses: 48.04; mean search slope for target present responses: 25.78; $F(1,116)=17.01$, $MSE=873.80$, $p<.001$; see Fig. S1 ). An interaction effect was also significant ($F(1,116)=8.91$, $MSE=873.80$, $p=.003$). These results match previous reports of steeper search slopes for searching a circle among circles crossed by a line than for the inverse search (e.g., Treisman & Souther, 1985).


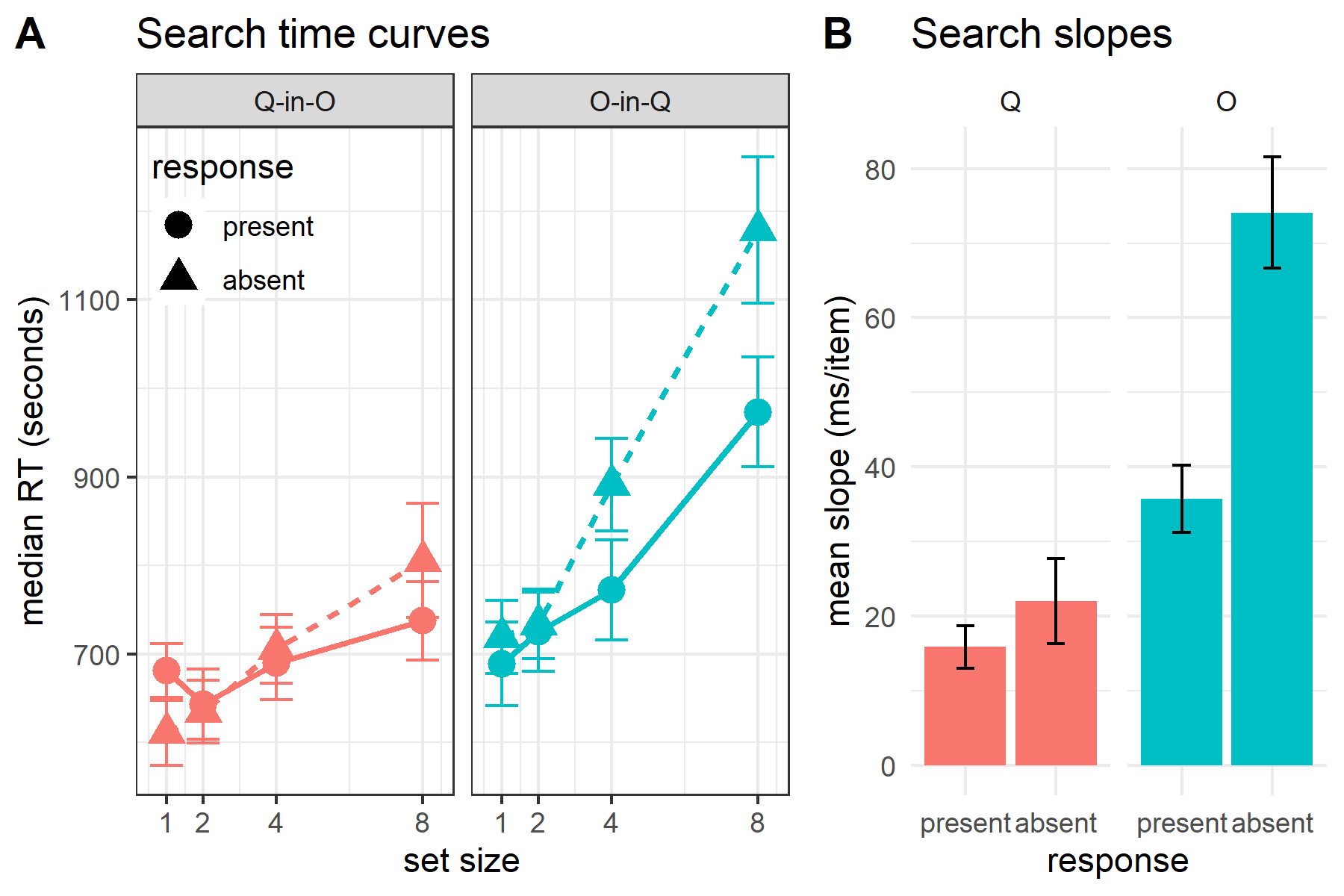


*Figure* *S1:* A: Median search time by distractor set size for the two search tasks and two responses. Correct responses only. Error bars represent the standard error of the median. B: mean search slope per target (Q or O) and response (present or absent). Error bars represent the standard error of the mean.

## Discrimination task: metacognitive asymmetry

Mean accuracy in the discrimination task was 0.76 ($M=0.76$, 95% CI $[0.69$, $0.83]$). mean SOA in the 32th trial was $M=22.54$, 95% CI $[16.23$, $28.85]$. Participants showed no consistent bias in their responses ($M=0.00$, 95% CI $[-0.04$, $0.03]$). On a scale of 0 to 1, mean confidence level was 0.53 ($M=0.53$, 95% CI $[0.36$, $0.70]$). Confidence was higher for correct than for incorrect responses ($M_{d}=0.15$, 95% CI $[0.09$, $0.21]$, $t(29)=5.24$, $p<.001$).

*Hypothesis 1*: In line with our hypothesis, confidence was generally higher for *Q* (feature present) responses than for *O* (feature absent) responses ($M_{d}=0.10$, 95% CI $[0.05$, $0.16]$, $t(29)=3.63$, $p=.001$; Cohen’s d = 0.66).

*Hypothesis 2*: In order to measure metacognitive asymmetry, we extracted the response-conditional type-2 ROC (rc-ROC) curves for the two responses (*Q* and *O*) in the discrimination task. This was done by plotting the cumulative distribution of confidence ratings (high to low) for correct responses against the same distribution for incorrect responses. The area under the rc-ROC curve (auROC) was then taken as a measure of metacognitive sensitivity (Kanai et al., 2010; Meuwese et al., 2014). In line with our hypothesis, auROC for *Q* responses ($M=0.68$, 95% CI $[0.56$, $0.81]$) was higher than for *O* responses ($M=0.59$, 95% CI $[0.49$, $0.68]$; $t(10)=2.40$, $p=.037$; Cohen’s d = 0.73; see Fig. S2), mirroring the metacognitive asymmetry for detection judgments.

*Hypothesis 3*: Metacognitive asymmetry was significantly higher than what would be expected based on an equal-variance SDT model with the same response bias and sensitivity ($t(10)=2.70$, $p=.022$, Cohen’s d=0.81).

*Hypothesis 4*: In line with our hypothesis, *Q* responses were faster on average than *O* responses ($t(29)=-3.64$, $=.001$ ; Cohen’s d = 0.67).


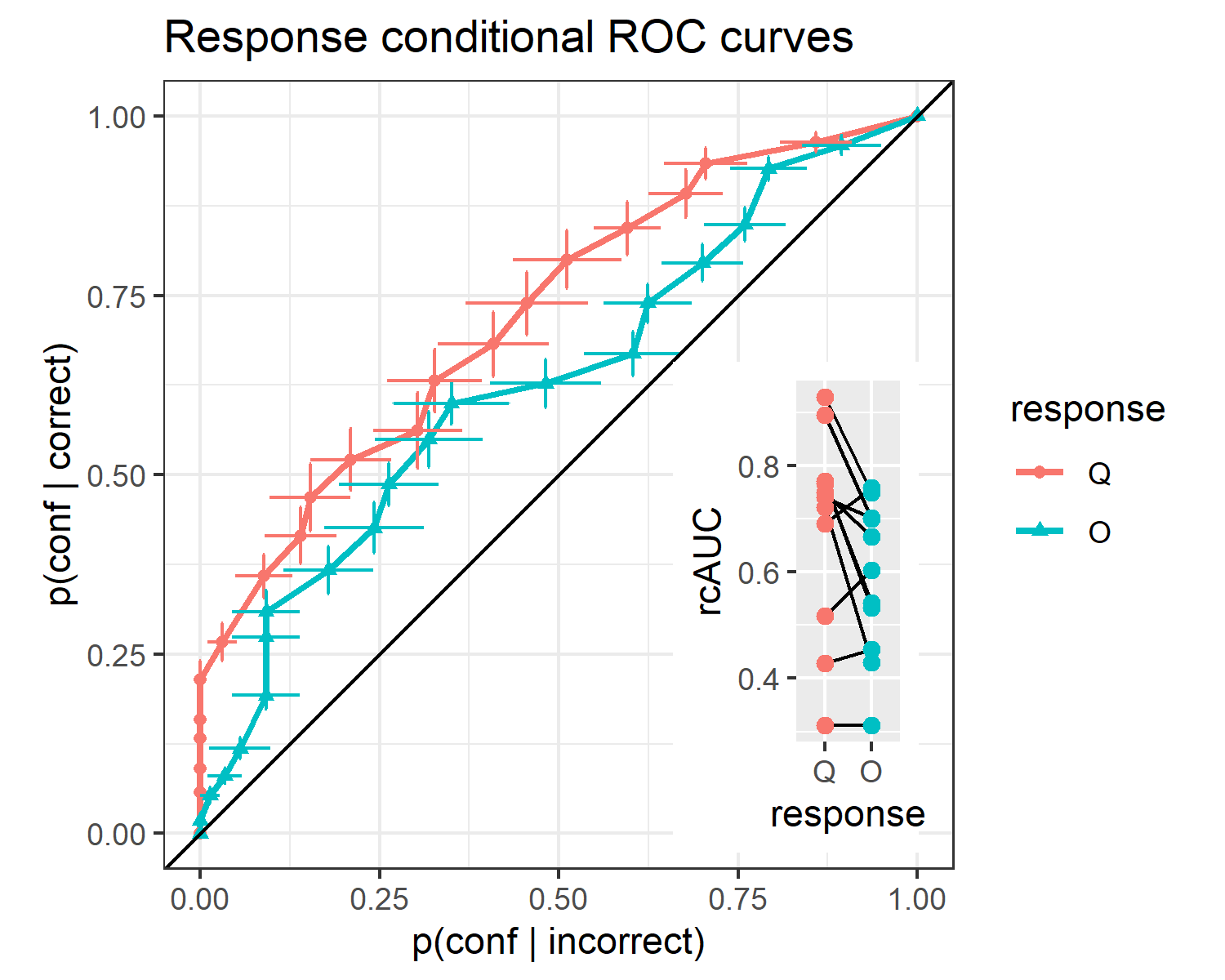


*Figure* *S2:* Response conditional ROC curves for the two discrimination responses. The area under the curve is a measure of metacognitive sensitivity. Bottom right inset: distributions of the area under the curve for the two responses, across participants. Overall, participants had lower metacognitive insight into the accuracy of their ‘O’ responses.

# References

Kanai, R., Walsh, V., & Tseng, C.-h. (2010). Subjective discriminability of invisibility: A framework for distinguishing perceptual and attentional failures of awareness. *Consciousness and Cognition*, *19*(4), 1045–1057.

Meuwese, J. D., Loon, A. M. van, Lamme, V. A., & Fahrenfort, J. J. (2014). The subjective experience of object recognition: Comparing metacognition for object detection and object categorization. *Attention, Perception, & Psychophysics*, *76*(4), 1057–1068.

Treisman, A., & Souther, J. (1985). Search asymmetry: A diagnostic for preattentive processing of separable features. *Journal of Experimental Psychology: General*, *114*(3), 285.
